# Generation of Werner-like states via a two-qubit system plunged in a thermal reservoir and their application in solving binary classification problems

**DOI:** 10.1038/s41598-021-82880-3

**Published:** 2021-02-11

**Authors:** E. Ghasemian, M. K. Tavassoly

**Affiliations:** Optics and Laser Group, Faculty of Physics, Yazd University, Yazd, Iran

**Keywords:** Optics and photonics, Physics

## Abstract

We present a theoretical scheme for the generation of stationary entangled states. To achieve the purpose we consider an open quantum system consisting of a two-qubit plunged in a thermal bath, as the source of dissipation, and then analytically solve the corresponding quantum master equation. We generate two classes of stationary entangled states including the Werner-like and maximally entangled mixed states. In this regard, since the solution of the system depends on its initial state, we can manipulate it and construct robust Bell-like state. In the continuation, we analytically obtain the population and coherence of the considered two-qubit system and show that the residual coherence can be maintained even in the equilibrium condition. Finally, we successfully encode our two-qubit system to solve a binary classification problem. We demonstrate that, the introduced classifiers present high accuracy without requiring any iterative method. In addition, we show that the quantum based classifiers beat the classical ones.

## Introduction

Realistic quantum systems are interesting and important due to their fundamental applications in up-to-date quantum technologies. Indeed, they are open systems where their inevitable interactions with surrounding environments are of statistical nature^1,2^. The study of composite open quantum systems is essential in quantum information and quantum computation tasks^3^. In this line, quantum entanglement and coherence are fundamental concepts in quantum mechanics and play the key role in quantum information processing^4–9^. Thus, the generation, control and protection of entanglement and coherence are critical in the context of open quantum systems. Accordingly, many researchers have focused on the study of open quantum systems in nonequilibrium environments^10–13^. The entanglement and coherence can be maintained in nonequilibrium steady states, whenever the nonequilibrium environments constantly exchange matter, energy and information with the quantum system^14–18^. The open quantum systems and their nonequilibrium steady state features open new doors for the generation and manipulation of quantum resources. In modern quantum information, the entanglement provides the basis underlying future quantum computers^19^ and quantum teleportation^5,20^. Hence, the quest for the pure maximally entangled states is somewhat counterbalanced by the growing appearance of the mixed states and non maximally entangled states in real physical systems. In recent years, within the investigations toward the characterization of the mixed states as a practical resource, a great deal of efforts has been done for preparing novel positive maps in Hilbert spaces in view of the assessment of residual entanglement and the establishment of new separability criteria for entangled states^21,22^. A wide range and consistent exploration of these aspects requires an available universal and flexible source for engineering of two-qubit photon states of any structure in a reliable and reproducible way.

In this line, the high brilliance spontaneous parametric down conversion has been previously implemented for synthesize the tunable Werner states as well as maximally entangled mixed states (MEMSs)^23^. Indeed, these states are key components used in modern quantum information due to the fact that their entanglement cannot be increased by any unitary transformation^24–27^. Among the well-known entangled states, Werner states also possess this property; hence, they have been classified as the generalized MEMSs^28^.

So far, the two-qubit systems have been extensively investigated for different aims with various approaches, specially for the generation of entangled states. For instance, the possibility of entanglement generated by the quantum reservoir during the Markovian regime through a purely noisy mechanism has been discussed in^29^. In^30^, the protection of entanglement via the quantum Zeno effect has been studied wherein the two qubits undergo the effect of local reservoirs. The authors in^31^ have investigated a system of qubits and higher dimensional spins interacting only through their mutual couplings to a reservoir. Nevertheless, such systems are still rich and some new aspects of them need to be further investigated for manifestation of new practical applications. Accordingly, we are motivated to investigate the possibility of using two-qubit systems for solving the decision problems related to machine learning context, as a subfield of artificial intelligence.

Nowadays, neural networks inspired from the brain structure are considered as essential tools for solving tasks which cannot be solved by traditional (classical) algorithms^32^. It has been proven that the quantum algorithms systematically outperform their classical counterparts^33,34^. For instance, Shor’s algorithm implemented on a trapped-ion quantum computer^35^ can be used to solve a particular class of non-deterministic polynomial hard problems via quantum annealing^36^. It is of fundamental interest to look for the advantages of quantum effects in neural network computing. The conceptual problem is that the dynamics of closed quantum systems is deterministic, while the dynamical evolution of neural networks is always dissipative which prevents any straightforward generalization of quantum neural networks computing^37^. The authors in^38^ proposed a framework based on open quantum systems to overcome this obstacle. They implemented a neural network using Markovian open quantum system where its dynamics was described by the Lindblad equation (density matrix operator approach). Also, the dissipative quantum computation as a reasonable platform has successfully been introduced for stable quantum neural network states^37^. In fact, the dissipative systems reach steady states during the interaction with quantum reservoirs and provide another new direction for quantum computation^2,39^. Mathematically speaking, a data classifier is the simplest neural network unit which connects input nodes to an output node^40^. For instance, support vector machine type classifiers are able to process purely classical data and use the state space to achieve the quantum advantage. Such classifiers map the data to a quantum state $\Phi : {\overrightarrow {x}} \in \Omega \longrightarrow {|{\Phi (\overrightarrow {x})}\rangle }{\langle {\Phi (\overrightarrow {x})}|}$, nonlinearly, where $\Omega \subset {\mathbb {R}}^d$^41^. It is worth to note that the density matrix $\rho (\overrightarrow{x})={|{\Phi (\overrightarrow {x})}\rangle }{\langle {\Phi (\overrightarrow {x})}|}$ which is considered as a “quantum software state”^42^ can be utilized to construct quantum algorithm for higher-level machine learning.

Based on the valuable works done toward new approaches to quantum computing, we motivated to implement an open two-qubit system to solve binary classification. In this paper we start with the generation of stationary entangled states of a two-qubit system with no direct interaction, embedded in a thermal bath as the source of dissipation. In this regard, at first, we solve the corresponding master equation of the two-qubit system under the influence of a global environment and we find its solution at steady state regime, while we consider a general initial two-qubit state. Then, we proceed to generate the desired entangled states. Results show that the steady state solutions are in the form of Werner-like and maximally entangled states. In addition, we manipulate the initial state of the system to generate robust Bell states. At last, as a new application of the system under study and as our second goal, we use the obtained steady state (solution) of two-qubit system to solve a binary classification problem i.e., we classify the vertebrates into two mammal and non-mammal groups, with enough high accuracy.

The contents of this paper are organized as follow. In next section, we introduce a dissipative model for our two-qubit system, solve the corresponding quantum master equation and then generate our desired entangled states. In third section, we investigate the steady state population and coherence of the system in the equilibrium condition. In the continuation, the steady state solution of two-qubit is used to solve binary classification in fourth section. Finally, last section includes a brief summary and our conclusions.

## The model and its solution

### The master equation of two-qubit system

**Figure 1:**
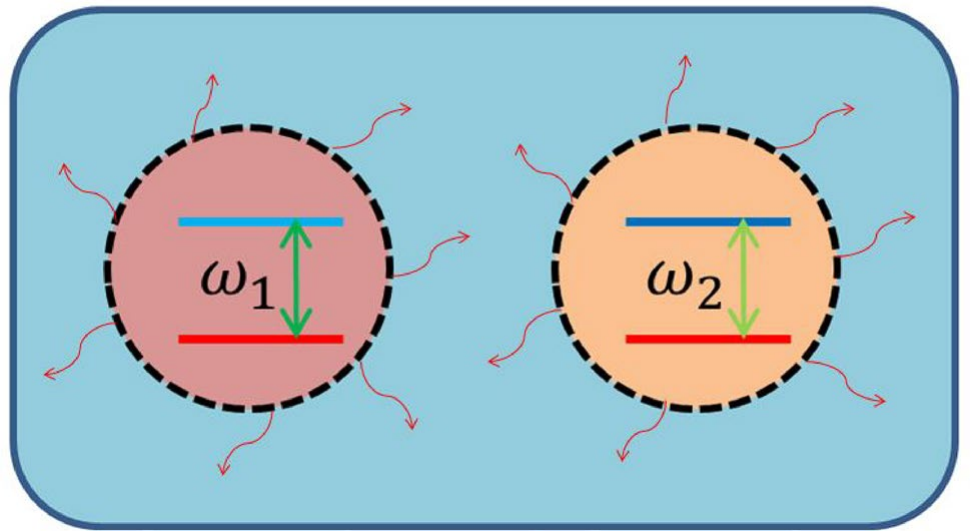
Schematic of two-qubit system undergoing dissipative dynamics via the interaction with a common thermal environment.

Open quantum systems undergo dissipation of energy into their environment. Usually, the environment is treated as a distribution of the uncorrelated thermal equilibrium mixture of states. The total Hamiltonian of a system consisting of two independent qubits, both embedded in a common thermal reservoir reads as,

1 $$\begin{aligned} {\hat{H}}_{\mathrm{T}}= & {} {\hat{H}}_{\mathrm{S}}+{\hat{H}}_{\mathrm{R}}, \nonumber \\ {\hat{H}}_{\mathrm{S}}= & {} \sum _{i=1}^{2}\omega _i \sigma _i^{\dagger } \sigma _i, \nonumber \\ {\hat{H}}_{\mathrm{R}}= & {} \sum _{k=0}^{\infty }\nu _k {\hat{b}}_k^{\dagger }{\hat{b}}_k, \end{aligned}$$

where ${\hat{H}}_{\mathrm{S}}$ and ${\hat{H}}_{\mathrm{R}}$ denote the Hamiltonians of the system and reservoir, respectively. Also, $\omega _i$ and $\sigma _i={|{g_i}\rangle }{\langle {e_i}|}$ are the frequency and lowering operator corresponding to the *i*th qubit, respectively, where ${|{e_i}\rangle }$ and ${|{g_i}\rangle }$ denote the excited and ground states, respectively. In addition, $\nu _k$ and ${\hat{b}}_k$ are the frequency and the annihilation operator corresponding to the *k*th mode of the thermal reservoir, respectively. The system is schematically shown in Fig. 1. Although, we do not consider direct interaction between the two qubits, they commonly interact with thermal reservoir that can be described by the following Hamiltonian,

2 $$\begin{aligned} {\hat{H}}_{\mathrm{I}}=\sum _{k=0}^{\infty } (\alpha _1 \sigma _1 + \alpha _2 \sigma _2){\hat{b}}_k^{\dagger }+ H.c, \end{aligned}$$

where $\alpha _i$ introduces the coupling constant of the reservoir with *i*th qubit for $i=1,2$.

Hence, the dynamics of system can be described by the master equation of Lindblad form^2,43^,

3 $$\begin{aligned} {\dot{\rho }}(t)= & {} {\mathcal {L}} \rho (t) \nonumber \\= & {} {\bar{n}}[2 (\sigma _1+\sigma _2) \rho (\sigma _1+\sigma _2)^\dag - (\sigma _1+\sigma _2)^\dag (\sigma _1+\sigma _2)\rho -\rho (\sigma _1+\sigma _2)^\dag (\sigma _1+\sigma _2)]\nonumber \\&\quad +({\bar{n}}+1) [2(\sigma _1+\sigma _2)^\dag \rho (\sigma _1+\sigma _2)- (\sigma _1+\sigma _2) (\sigma _1+\sigma _2)^\dag \rho -\rho (\sigma _1+\sigma _2) (\sigma _1+\sigma _2)^\dag ], \end{aligned}$$

where ${\mathcal {L}}$ denotes the Lindblad operator and ${\bar{n}}$ is the mean number of thermal excitations corresponds to the thermal bath, i.e., the source of dissipation in open quantum systems. (The details of derivation of the Lindblad equation corresponding to the interaction between two-qubit systems and their environment can be found in^44^). One can formally expand the density operator in the following basis,

4 $$\begin{aligned} \{|1\rangle := |e_1\rangle |e_2\rangle ; \quad |2\rangle := |e_1\rangle |g_2\rangle ; \quad |3\rangle := |g_1\rangle |e_2\rangle ; \quad |4\rangle := |g_1\rangle |g_2\rangle \}. \end{aligned}$$

So the density operator of the two-qubit system can be rewritten as,

5 $$\begin{aligned} \rho (t) = \sum _{j,k=1}^4 \rho _{j,k}(t) |j\rangle \langle k|, \end{aligned}$$

where $\rho _{j,k}(t)$ are unknown time-dependent coefficients. Upon inserting (5) into (3), the dynamics of system is described by a set of linear differential equations for the unknown coefficients $\rho _{j,k}(t)$ that can be compactly expressed as,

6 $$\begin{aligned} \dot{{\mathbf {v}}}(t) = M{{\mathbf {v}}}(t), \end{aligned}$$

where

$$\begin{aligned} {{\mathbf {v}}}(t) = \left( \rho _{11}(t), \rho _{12}(t), \ldots , \rho _{43}(t), \rho _{44}(t)\right) ^\top , \end{aligned}$$

and *M* is a $16\times 16$ matrix of constant coefficients which is explicitly presented in Appendix A.

### Generation of entangled states: steady state solution of the master equation

So far, the two-qubit systems have extensively been explored with different schemes (^11–13^ and Refs. therein). For instance, we recently investigated the dynamical evolution of such system ( see Ref.^43^). In this regard, however, we should emphasize that in the present paper we intend to focus on the steady state solutions of the system due to the reasons which will be clear in the continuation of the paper. Also, the stationary entanglement (and Bell violation) of a two-qubit system collectively interacting with a common thermal reservoir was discussed in^45^. Although, the system considered in^45^ is the same as ours, but there exist differences which we briefly mention. As th first case, the authors in^45^ solved the master equation by considering Werner-like state as initial state, while we proceed to find the solution with a general two-qubit state including the Werner and Werner-like states. In addition, one should notice that, when the dimension of Lindblad operator is greater than one, the steady state of system is not unique and depends on the initial state. In this case, the solution must be obtained carefully since the excitation number and the elements of initial density matrix strongly affect the solution of master equation. In particular, the system should be analyzed distinctly in zero and non-zero temperatures. Indeed, the Lindblad operator may possess different dimensions at zero temperature (${\bar{n}} = 0$) with respect the nonzero temperatures (${\bar{n}} \ne 0$). As result, it is necessary to follow up this distinction as we do in the continuation of the paper. Moreover, it is worth to note that finding the solution of the system with a general initial state is mandatory in order to solve the classification problem, since we want to use the elements of initial (steady) states of the system as the inputs (outputs) of classification problem.

Unfortunately the steady state of the considered two-qubit system cannot be found by solving the equation $M{{\mathbf {v}}}(t=\infty )=0$, since the dimension of $\ker (M)$ is greater than one, meaning that the steady state is not unique and depends on the initial state of the two-qubit system (see Appendix A). This should be ascribed to the fact that in Eq. (3) there exist non-trivial operators (i.e. not multiple of the identity) commuting with the Lindblad operators^46^. As a consequence, the steady states must be derived by first solving Eq. (6) with given initial conditions and then taking $\lim _{t\rightarrow \infty } {{\mathbf {v}}}(t)$. In this line, we consider a general two-qubit density matrix as the initial state of the system and solve the corresponding set of coupled linear differential equations. Since the general time-dependent solution of Eq. (6) is too cumbersome, we just present the analytical solution for ${\bar{n}}=0$ in Appendix B. At this point, we want to generate stationary entangled state, therefore, we pay our attention to find the steady state solutions of system.

In the case of vacuum reservoir i.e., ${\bar{n}}=0$ and by considering an arbitrary general state for two-qubit $\rho (0)$ as blow, the steady state solution $\rho (\infty )$ reads as,

7 $$\begin{aligned} \rho (0)=\begin{bmatrix} \rho _{11} &{} \rho _{12} &{} \rho _{13} &{} \rho _{14}\\ \rho _{21} &{} \rho _{22}^\diamond &{} \rho _{23}^\diamond &{} \rho _{24}^\diamond \\ \rho _{31} &{} \rho _{32}^\diamond &{} \rho _{33}^\diamond &{} \rho _{34}^\diamond \\ \rho _{41} &{} \rho _{42}^\diamond &{} \rho _{43}^\diamond &{} \rho _{44}\\ \end{bmatrix},\qquad \rho (\infty )= \begin{bmatrix} 0 &{} 0 &{} 0 &{} 0\\ 0 &{} P_1 &{} -P_1 &{} P_2\\ 0 &{} -P_1 &{} P_1 &{} -P_2\\ 0 &{} P_2^* &{} -P^*_2 &{} P_3\\ \end{bmatrix}, \end{aligned}$$

where

8 $$\begin{aligned} P_1= & {} \frac{1}{4}(\rho _{22}+\rho _{33}-\rho _{23}-\rho _{32}),\nonumber \\ P_2= & {} \frac{1}{2}(\rho _{24}-\rho _{34}). \end{aligned}$$

The last element of the steady state of system clearly satisfies $P_3=1-2P_1$. It should be noted that the stationary solutions of system in this condition depends only on those elements of initial density matrix which are marked with the square symbol ($\diamond$).

Assuming $\rho _{24}=\rho _{34}$ ($P_2=0$), we arrive at,

9 $$\begin{aligned} \rho _{\mathrm{MEMS}}= \begin{bmatrix} 0 &{} 0 &{} 0 &{} 0\\ 0 &{} P_1 &{} -P_1 &{} 0\\ 0 &{} -P_1 &{} P_1 &{} 0\\ 0 &{} 0 &{} 0 &{} P_3\\ \end{bmatrix}, \end{aligned}$$

which represents a MEMS and can be rewritten as $\rho _{ \mathrm MEMS}=2P_1 {|{\Psi }\rangle }{\langle {\Psi }|}+P_3 {|{4}\rangle }{\langle {4}|}$, where ${|{\Psi }\rangle }=\frac{1}{\sqrt{2}}({|{2}\rangle }-{|{3}\rangle })$ is pure singlet (Bell) state. Indeed, the explicit form of this Bell state can be rewritten in terms of the bare basis of the two-qubit system as ${|{\Psi }\rangle }=\frac{1}{\sqrt{2}}({|{e_1,g_2}\rangle }-{|{g_1,e_2}\rangle })$. Interestingly, Eq. (8) implies that for $P_1=\frac{1}{2}$ and $P_2=P_3=0$, the initial and final states of system are the same Bell states. Indeed, we can state that the system undergoes a transformation as,

10 $$\begin{aligned} \rho (0)=\begin{bmatrix} 0 &{} 0 &{} 0 &{} 0\\ 0 &{} \rho _{22} &{} \rho _{23} &{} 0\\ 0 &{} \rho _{32} &{} \rho _{33} &{} 0\\ 0 &{} 0 &{} 0 &{} 0\\ \end{bmatrix}, \qquad \rho (\infty )= \begin{bmatrix} 0 &{} 0 &{} 0 &{} 0\\ 0 &{} P_1 &{} -P_1 &{} 0\\ 0 &{} -P_1 &{} P_1 &{} 0\\ 0 &{} 0 &{} 0 &{} 0\\ \end{bmatrix}. \end{aligned}$$

Therefore, although the initial state of system undergoes time evolution during the interaction, nevertheless it can be recovered at the steady state regime. In other words, once the two-qubit system interacts with its environment (vacuum reservoir), the two qubits may reconstruct their initial state i.e., a robust Bell state can be generated at steady state regime.

In the other hand, the steady state solution of system with ${\bar{n}}>0$, by assuming a general initial two-qubit state, can be obtained as,

11 $$\begin{aligned} \rho (0)= \begin{bmatrix} \rho _{11}^\star &{} \rho _{12} &{} \rho _{13} &{} \rho _{14}\\ \rho _{21} &{} \rho _{22}^\star &{} \rho _{23}^\star &{} \rho _{24}\\ \rho _{31} &{} \rho _{32}^\star &{} \rho _{33}^\star &{} \rho _{34}\\ \rho _{41} &{} \rho _{42} &{} \rho _{43} &{} \rho _{44}^\star \\ \end{bmatrix},\qquad \rho (\infty )= \begin{bmatrix} Q_1 &{} 0 &{} 0 &{} 0\\ 0 &{} Q_2 &{} Q_3 &{} 0\\ 0 &{} Q_3 &{} Q_2 &{} 0\\ 0 &{} 0 &{} 0 &{} Q_4\\ \end{bmatrix}, \end{aligned}$$

where $Q_i$ for $i=1,2,3$ are given as follow,

12 $$\begin{aligned} Q_1= & {} \rho _{11}(\infty )=\frac{f_{11}({\bar{n}})}{g_1({\bar{n}})}(\rho _{11}+\rho _{44})+\frac{f_{12}({\bar{n}})}{g_1({\bar{n}})}(\rho _{22}+\rho _{33}+\rho _{23}+\rho _{32}), \end{aligned}$$

13 $$\begin{aligned} Q_2= & {} \rho _{22}(\infty )=\frac{f_{21}({\bar{n}})}{g_2({\bar{n}})}(\rho _{11}+\rho _{44}))+\frac{f_{22}({\bar{n}})}{g_2({\bar{n}})}(\rho _{22}+\rho _{33})+\frac{f_{23}({\bar{n}})}{g_2({\bar{n}})}(\rho _{23}+\rho _{32}), \end{aligned}$$

14 $$\begin{aligned} Q_3= & {} \rho _{23}(\infty )=\frac{f_{31}({\bar{n}})}{g_2({\bar{n}})}(\rho _{11}+\rho _{44}))+\frac{f_{32}({\bar{n}})}{g_2({\bar{n}})}(\rho _{22}+\rho _{33})+\frac{f_{33}({\bar{n}})}{g_2({\bar{n}})}(\rho _{23}+\rho _{32}), \end{aligned}$$

whit $f_{i,j}({\bar{n}})$ and $g_k({\bar{n}})$ which are given in Appendix C. It should be noted that $Q_4$ can be obtained as $Q_4=1-Q_1-2Q_2$. Eqs. (12)-(14) reveal that the effective initial state of the two-qubit just depend on the elements which marked with the star symbol ($\star$) (see $\rho (0)$ in Eq. (11)). These elements define the well-known Werner-like entangled states. It is worthwhile to note that Werner states and MEMS are two particular classes of mixed states whose their density matrices in the previously explained basis $\{{|{1}\rangle },{|{2}\rangle },{|{3}\rangle },{|{4}\rangle }\}$ possess the same form as the stationary solution of our two-qubit system ($\rho (\infty )$ in Eq. (11)). The Werner state $\rho _W= p{|{\Psi }\rangle }{\langle {\Psi }|}+(1-p)\frac{\mathbf{I}_4}{4}$ is a mixture of a pure singlet state ${|{\Psi }\rangle }=\frac{1}{\sqrt{2}}({|{2}\rangle }-{|{3}\rangle })$ with probability *p* and a fully mixed state with probability $1-p$ ($0\le p \le 1$) which can be expressed by the unit operator defined in the 4-dimensional Hilbert space ${\mathbf{I}}_4$. The density matrix of Werner state can also be rewritten as,

15 $$\begin{aligned} \rho _W= \begin{bmatrix} \frac{1-p}{4} &{} 0 &{} 0 &{} 0\\ 0 &{} \frac{1+p}{4} &{} \frac{-p}{2} &{} 0\\ 0 &{} \frac{-p}{2} &{} \frac{1+p}{4} &{} 0\\ 0 &{} 0 &{} 0 &{} \frac{1-p}{4}\\ \end{bmatrix}. \end{aligned}$$

Based on the singlet weight *p*, Werner states may be classified as entangled ($p > \frac{1}{3}$) and separable states ($p \le \frac{1}{3}$). Moreover, the Werner states do not violate the Bell inequality in the range $\frac{1}{3}< p < \frac{1}{\sqrt{2}}$ in spite of being nonseparable entangled states; more precisely, they are negative partial transpose states^21,47,48^. Also, the Werner states are of fundamental interest in quantum information due to the fact that they demonstrate a decoherence process occurring on a singlet state traveling along a noisy channel^1^.

The steady state solution of our considered system given by Eq. (11) introduces a class of extended Werner-like states. Hence, one can easily find out that how to manipulate the initial state of system to generate a robust entangled two-qubit state. Therefore, as a key point, it should be noted that once an open two-qubit system is driven with an initial Werner-like state, it may reconstruct a stationary Werner-like state in the steady state regime. For the sake of simplicity let us consider the case of ${\bar{n}}=1$ in which we arrive at the following quantities,

16 $$\begin{aligned} Q_1= & {} \rho _{11}(\infty )=\frac{1}{14}(\rho _{22}+\rho _{33}+\rho _{23}+\rho _{32})+\frac{1}{7}(\rho _{11}+\rho _{44}), \nonumber \\ Q_2= & {} \rho _{22}(\infty )=\frac{1}{28}(9[\rho _{22}+\rho _{33}]-5[\rho _{23}+\rho _{32}])+\frac{1}{7}(\rho _{11}+\rho _{44}), \nonumber \\ Q_3= & {} \rho _{23}(\infty )=\frac{1}{28}(-5[\rho _{22}+\rho _{33}]+9[\rho _{23}+\rho _{32}])+\frac{1}{7}(\rho _{11}+\rho _{44}), \nonumber \\ Q_4= & {} \rho _{44}(\infty )=\frac{2}{7}(\rho _{22}+\rho _{33}+\rho _{23}+\rho _{32})+\frac{4}{7}(\rho _{11}+\rho _{44}), \end{aligned}$$

where the normalization condition $Q_1+2Q_2+Q_4=\rho _{11}+\rho _{22}+\rho _{33}+\rho _{44}=1$ is satisfied.

Now, let us use Eq. (15) and construct a Werner state as the initial state of system. For $p=1$, we arrive at ${|{\Psi }\rangle }=\frac{1}{\sqrt{2}}({|{2}\rangle }-{|{3}\rangle })$ which is a special Werner-like (Bell) state. Under this condition, we have $Q_1=Q_4=0$ and $Q_2=-Q_3=\frac{1}{2}$, therefore the stationary solution of two-qubit system is a Werner-like (Bell) state. Thus, we can state that a dissipative two-qubit driven with initial Bell state may reconstruct its original state as time passes and the system approaches its stationary state. For $p=0$ the initial and stationary states of system can be obtained as below,

17 $$\begin{aligned} \rho (0)= \begin{bmatrix} \frac{1}{4} &{} 0 &{} 0 &{} 0\\ 0 &{} \frac{1}{4} &{} 0 &{} 0\\ 0 &{} 0 &{} \frac{1}{4} &{} 0 \\ 0 &{} 0 &{} 0 &{} \frac{1}{4}\\ \end{bmatrix},\qquad \rho (\infty )= \begin{bmatrix} \frac{3}{28} &{} 0 &{} 0 &{} 0\\ 0 &{} \frac{13}{56} &{} \frac{-1}{56} &{} 0\\ 0 &{} \frac{-1}{56} &{} \frac{13}{56} &{} 0\\ 0 &{} 0 &{} 0 &{} \frac{3}{7}\\ \end{bmatrix}. \end{aligned}$$

As is clear, the chosen initial state is fully mixed state (${\mathbf{I}}_4$) and the final state is a Werner-like state. Interestingly, once the initial state of system is the separable state $\rho (0)={|{1}\rangle }{\langle {1}|}$, the stationary solution of system is a Werner-like state which consists of a Bell state ${|{\Phi }\rangle }=\frac{1}{\sqrt{2}}({|{2}\rangle }+{|{3}\rangle })$ and a mixture of basis ${|{1}\rangle }$ and ${|{4}\rangle }$ which can be given as below,

18 $$\begin{aligned} \rho (\infty )= \begin{bmatrix} \frac{1}{7} &{} 0 &{} 0 &{} 0\\ 0 &{} \frac{1}{7} &{} \frac{1}{7} &{} 0\\ 0 &{} \frac{1}{7} &{} \frac{1}{7} &{} 0\\ 0 &{} 0 &{} 0 &{} \frac{4}{7}\\ \end{bmatrix}. \end{aligned}$$

### Concurrence of the two-qubit system

For a mixed state represented by the density operator $\rho$, one can define a spin-flip operation as below^49^,

19 $$\begin{aligned} {\tilde{\rho }}=(\sigma _y^1 \otimes \sigma _y^2) \rho ^* (\sigma _y^1 \otimes \sigma _y^2), \end{aligned}$$

where $\rho ^*$ is the complex conjugate of the density matrix of the two qubits $\rho$ and $\sigma _y$ is the Pauli matrix. Now, by considering $R:=\rho \cdot {\tilde{\rho }}$ and using the square roots of the eigenvalues of the matrix *R* denoted by ${\mathcal {G}}_i$, the concurrence of the mixed state $\rho$ can be obtained as below,

20 $$\begin{aligned} C(\rho )=\max (0,{\mathcal {G}}_1-{\mathcal {G}}_2-{\mathcal {G}}_3-{\mathcal {G}}_4), \end{aligned}$$

where ${\mathcal {G}}_i>0$ and also ${\mathcal {G}}_1$ is the largest of them. Now, we want to investigate the entanglement dynamics of the two-qubit system by considering different initial states of the two qubits. Fortunately, the analytical expression of concurrence with initial Werner state can be derived as below^50^,

21 $$\begin{aligned} C({\rho _W})=2\max \{0, K_1,K_2\}, \qquad K_1=|\rho _{23}|-\sqrt{\rho _{11}\rho _{44}}, \qquad K_2=-\sqrt{\rho _{22}\rho _{33}}. \end{aligned}$$

**Figure 2:**
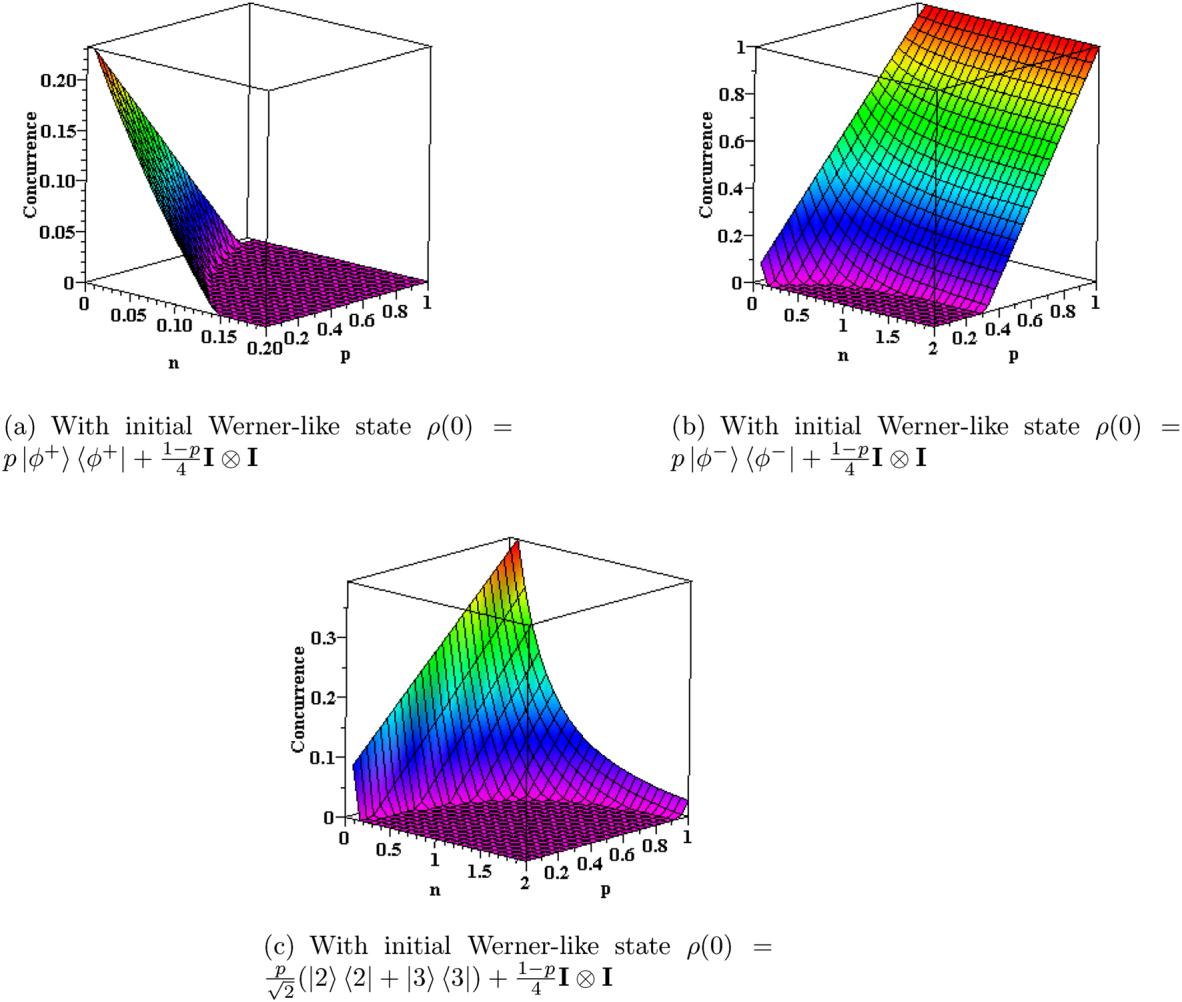
The concurrence of the two-qubit system for different initial states where ${|{\phi ^{\pm }}\rangle }=\frac{1}{\sqrt{2}}({|{2}\rangle } \pm {|{3}\rangle })$.

Figure 2 shows the steady state concurrence of the system for three different initial states at non-zero temperatures (${\bar{n}} \ne 0$). We can conclude that the amount of steady state entanglement strongly depends on the initial state of system. For instance, the system reaches the highest value of entanglement ($C=1$) in Fig. 2b, while in other plots, the system shows a relative maximum of entanglement at a single point. The death of entanglement (zero values of concurrence) is also clear in all plots of Fig. 2. Generally, the entanglement decreases by increasing the mean number of quanta of thermal reservoir, specially for small values of parameter *p*. Moreover, Fig. 2a demonstrates that the stationary state entanglement decreases with the increase of both parameters *p* and ${\bar{n}}$, which implies that the higher initial entanglement does not result in the higher stationary state entanglement.

## Equilibrium populations and coherence

The stationary solutions of the system clearly show that the diagonal elements (populations) of the density matrix are in general coupled with a pair of off-diagonal elements (coherence). For ${\bar{n}}>0$, the populations can be obtained by $Q_i$ (i=1,2,4) and the coherence can be extracted from $Q_3$. Recall that the off-diagonal elements of the initial state i.e., $\rho _{23}$ and $\rho _{32}$ demonstrate the initial coherence between the two qubits that gradually disappear due to the decoherence process^51^. In non-equilibrium condition, during the interaction, these elements still exist, while in the steady state (equilibrium) condition they vanish. Hence, for ${\bar{n}}>0$ and at equilibrium condition i.e., $\rho _{23}=\rho _{32}=0$, the populations corresponding to our two-qubit system can be obtained as,

22 $$\begin{aligned} W_{11}= & {} \rho _{11}(\infty )=\frac{f_{11}({\bar{n}})}{g_1({\bar{n}})}(\rho _{11}+\rho _{44})+\frac{f_{12}({\bar{n}})}{g_1({\bar{n}})}(\rho _{22}+\rho _{33}), \end{aligned}$$

23 $$\begin{aligned} W_{22}=W_{33}= & {} \rho _{22}(\infty )=\frac{f_{21}({\bar{n}})}{g_2({\bar{n}})}(\rho _{11}+\rho _{44})+\frac{f_{22}({\bar{n}})}{g_2({\bar{n}})}(\rho _{22}+\rho _{33}), \end{aligned}$$

where $f_{ij}({\bar{n}})$ for $i,j=1,2$ and $g_k({\bar{n}})$ with $k=1,2$ are defined in Appendix C. It should be noted that the population associated with the state $\rho _{44}(\infty )$ can be computed as $W_{44}= 1- (W_{11}+2W_{22})$. Also, the quantum coherence at equilibrium condition reads as,

24 $$\begin{aligned} C_{\mathrm{eq}}= & {} \frac{f_{31}({\bar{n}})}{g_2({\bar{n}})}(\rho _{11}+\rho _{44})+\frac{f_{32}({\bar{n}})}{g_2({\bar{n}})}(\rho _{22}+\rho _{33}). \end{aligned}$$

**Figure 3:**
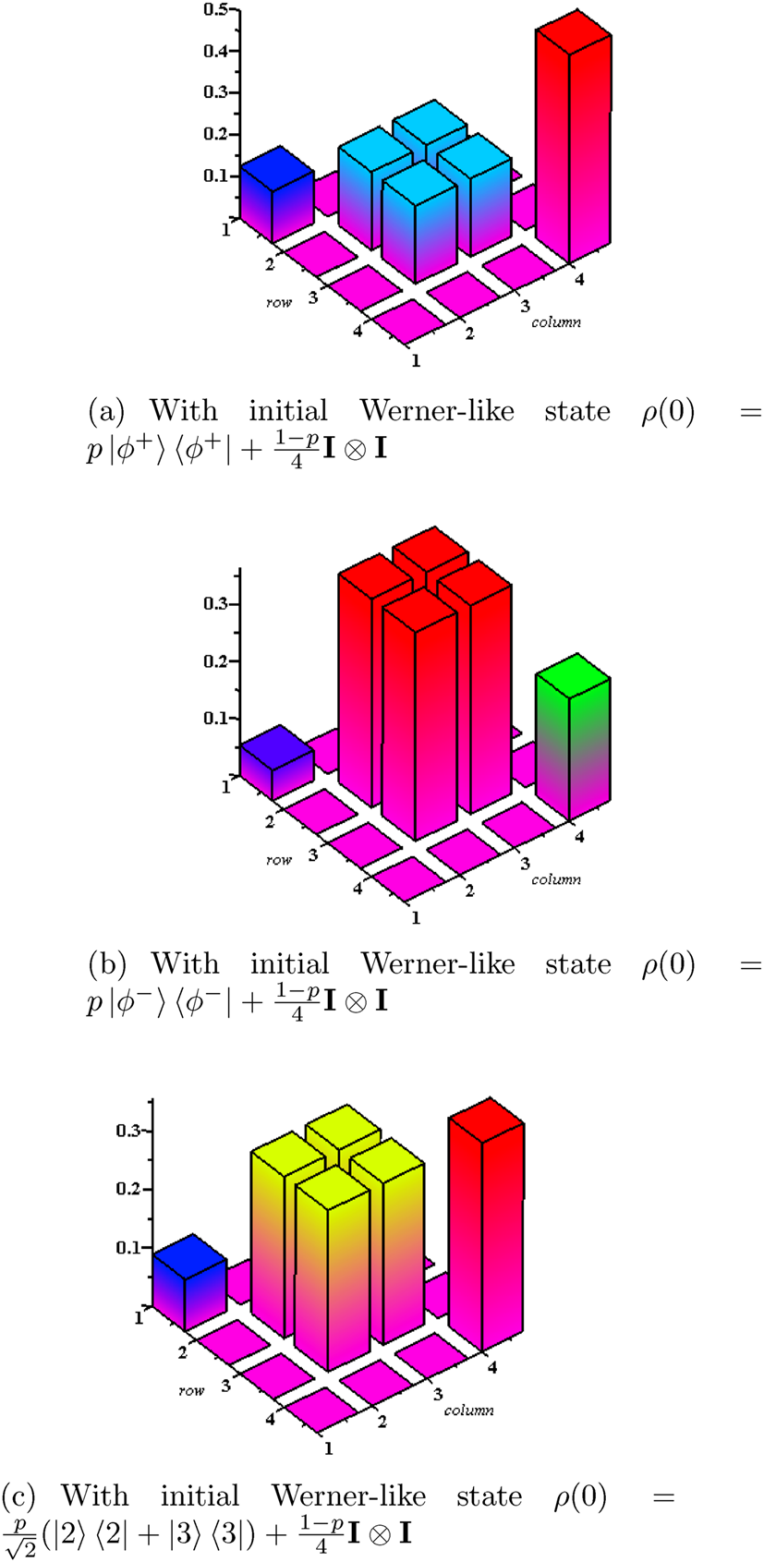
The population (diagonal elements) and coherence (nondiagonal elements) of the two-qubit system for ${\bar{n}}=1$ and ${|{\phi ^{\pm }}\rangle }=\frac{1}{\sqrt{2}}({|{2}\rangle } \pm {|{3}\rangle })$ with $p=0.5$ and considering different initial states.

Eq. (24) implies that in general quantum coherence does not vanish. Indeed, the interqubit interaction results in a residual quantum coherence even at equilibrium condition. Moreover, one can conclude that the generated entangled states in the previous section are robust against the decoherence process. For instance, the stationary solution at equilibrium condition for ${\bar{n}}=0$, can be obtained as below,

25 $$\begin{aligned} \rho _{\mathrm{eq}} (\infty )= \begin{bmatrix} 0 &{} 0 &{} 0 &{} 0\\ 0 &{} P_1 &{} -P_1 &{} 0\\ 0 &{} -P_1 &{} P_1 &{} 0\\ 0 &{} 0 &{} 0 &{} P_3\\ \end{bmatrix}, \end{aligned}$$

where $P_1=\frac{1}{4}(\rho _{22}+\rho _{33})$ and $P_3=1-2P_1$. Eq. (25) defines a class of robust MEMSs at equilibrium condition which can be manipulated by the diagonal elements of the initial two-qubit density matrix. Also, one can generate other classes of MEMSs and Werner-like states at equilibrium condition for ${\bar{n}}>0$ by using $\rho (\infty )$ in Eq. (11) and considering decoherence process ($\rho _{23}=\rho _{23}=0$) in Eqs. (12)–(14). Figure 3 depicts the evolution of population (diagonal elements) and coherence (nondiagonal elements) corresponding to the considered system at nonequilibrium and equilibrium conditions. Indeed, the first two plots show the density matrices of the system at nonequilibrium condition, while the last plot corresponds to the equilibrium regime. The nondiagonal elements of density matrix in equilibrium condition implies the residual coherence in the presence of dissipation. In should be noted that all plots clearly demonstrate the generation of Werner-like states. Also, the contributions of Bell states clearly can be observed in these plots.

## Binary classification via the stationary state of the dissipative two-qubit system

Classification is usually considered as a discipline in the context of machine learning, a subfield of artificial intelligence^52^. Indeed, classification, as a form of supervised learning, is a task of assigning objects to one of several predefined categories. It encompasses many diverse applications including detecting email messages based on message header or contents, categorizing cells as malignant or benign upon the results of MRI scans and so on. The input data for classification task is a collection of records. Each record is characterized by a tuple (*x*, *y*) where *x* denotes the initial attribute set and *y* is a special attribute which is considered as the class label (target attribute). Mathematically, classification is a task of learning a target function *f* that maps each attribute set *x* to one of predefined class labels *y*. The target function is also known as a classification model. A classification model can be considered as an explanatory tool to distinguish between objects of different classes. Also, it can be used to predict the class label of unknown records. A classifier requires a systematic procedure to perform a classification task from an input data set with binary or nominal categories. There are different classification techniques such as decision tree classifier, ruled-based classifiers, neural network, support vector machines and so on. Each classifier employs a learning algorithm to identify a model that presents the best relationship between the attribute set and the class label of the input data. The proposed model should be able to fit the input data and correctly predict the class labels of unknown records. The key objective of the learning algorithm is to built a classifier with high performance. Indeed, it should accurately predict the class label of previously unknown records. The details of basic concept of classification tasks can be found in literature^53,54^.

As is well-known, “any algorithmic process can be simulated efficiently using a probabilistic Turing machine”^1^. The quantum Turing machine model of computation has been shown to be equivalent to the model based upon quantum circuits. Besides these, we know that many computational problems can be formulated as decision problems; problems with a yes or no answer; for example, “Is a given number *m* a prime number or not?”, is a primality decision problem. Decision problems may be encoded in an obvious way as problems about languages. For instance, the primality decision problem can be encoded using the binary alphabet $\Sigma = \{0, 1\}$. To solve the primality decision problem, one requires a Turing machine which, when started with a given number *n* on its input tape, eventually returns “yes” if *n* is prime, and “no” if *n* is not prime. To make this process precise, one should slightly modify the definition of old Turing machine by replacing the halting state $q_h$ with two states $q_Y$ and $q_N$ to represent the answers “yes” and “no”, respectively.

More generally, a language *L* is decided by a Turing machine if the machine is able to decide whether an input *x* on its tape is a member of the language *L* or not. In other words, the machine halts in the state $q_Y$ if $x \in L$, and returns the state $q_N$ if $x \notin L$. Therefore, we can state that the machine has accepted or rejected *x* depending on which of these two cases comes about.

Now, it is worth to explain how the information can be transmitted and processed in real physical systems. The quantum noises are needed to understand the real-world quantum information processing and the quantum operations formalism, as a powerful mathematical tool for understanding quantum noise. The interaction of system with its surrounding environment leads to quantum noise. In theoretical quantum optics context, the quantum reservoirs are considered as the source of dissipation process (noise). Based on Shannon’s second fundamental theorem i.e., noisy channel coding theorem, one can quantify how much information can be reliably transmitted through a noisy communications channel. Such a channel has a finite input alphabet ${\mathcal {I}}$, and a finite output alphabet ${\mathcal {O}}$. For instance, the binary symmetric channel possesses identical input and output alphabets ${\mathcal {I}} = {\mathcal {O}} =\{0, 1\}$^1^. Here, we recall that the evolution of our considered system can be interpreted as a dissipative map (or noisy channel) $\rho (\infty )={\mathcal {D}}(\rho (0))$. Indeed, the following transformation may be found from Eq. (7),

26 $$\begin{aligned} \rho (\infty )=\sum _{j=1}^{4}K_{j}\rho (0)K_{j}^\dagger , \end{aligned}$$

where the corresponding Kraus operators $K_{j}$ can be obtained as,

27 $$\begin{aligned} K_1= & {} \frac{1}{2} \begin{bmatrix} 0 &{} 0 &{} 0 &{} 0\\ 0 &{} 1 &{} -1 &{} 0\\ 0 &{} -1 &{} 1 &{} 0 \\ 0 &{} 0 &{} 0 &{} 2\\ \end{bmatrix}, \nonumber \\ K_2= & {} \begin{bmatrix} 0 &{} 0 &{} 0 &{} 0\\ 0 &{} 0 &{} 0 &{} 0\\ 0 &{} 0 &{} 0 &{} 0 \\ 1 &{} 0 &{} 0 &{} 0\\ \end{bmatrix}, \nonumber \\ K_3= & {} \begin{bmatrix} 0 &{} 0 &{} 0 &{} 0\\ 0 &{} 0 &{} 0 &{} 0\\ 0 &{} 0 &{} 0 &{} 0 \\ 0 &{} 0 &{} 0 &{} 0\\ \end{bmatrix}, \nonumber \\ K_4= & {} \frac{1}{\sqrt{2}} \begin{bmatrix} 0 &{} 0 &{} 0 &{} 0\\ 0 &{} 0 &{} 0 &{} 0\\ 0 &{} 0 &{} 0 &{} 0 \\ 0 &{} 1 &{} 1 &{} 0\\ \end{bmatrix}. \end{aligned}$$

Such transformations are frequently used in quantum computation task. Also, it is possible to design a quantum circuit to preform such transformations^1^. (The details of such an approach can be found in related literature. For instance some useful examples and exercises can be found in^1^).

For instance, being able to realize arbitrary unitary transformations for a spin system coupled with RF pulses is one of the most attractive aspects of “nuclear magnetic resonance” for quantum computation^1^. Here, it is worth to note that a classical computer is analyzed by measuring its internal state at different points in time. While, for a quantum computer, an essential technique i.e., “state tomography” is used to measure its density matrix^1^. Now, as a practical application of open quantum systems, we are interested to solve a binary classification problem with the help of the stationary solution of the two-qubit system. Indeed, we would like to classify the vertebrates into two categories: mammals and non-mammals. For this purpose, we require a training set consisting of records whose class labels are known and a test set which consists of records with unknown class labels. The training set is used to construct a classification model and subsequently applied to the test set.

**Table 1:** The training data set corresponding to vertebrates extracted from: https://docplayer.net/4264600-Classification-basic-concepts-decision-trees-and-model-evaluation.html.

Record	Body temperature	Give birth	Has legs	Hibernates	Aquatic	Aerial	Class label
Human	Warm-blooded	Yes	Yes	No	No	No	Mammal
Python	Cold-blooded	No	No	Yes	No	No	Reptile
Salmon	Cold-blooded	No	No	No	Yes	No	Fish
Whale	Warm-blooded	Yes	No	No	Yes	No	Mammal
Frog	Cold-blooded	No	Yes	Yes	Semi	No	Amphibian
Komodo dragon	Cold-blooded	No	Yes	No	No	No	Reptile
Bat	Warm-blooded	Yes	Yes	Yes	No	Yes	Mammal
Pigeon	Warm-blooded	No	Yes	No	No	Yes	Bird
Cat	Warm-blooded	Yes	Yes	No	No	No	Mammal
Leopard shark	Cold-blooded	Yes	No	No	Yes	No	Fish
Turtle	Cold-blooded	No	Yes	No	Semi	No	Reptile
Penguin	Warm-blooded	No	Yes	No	Semi	No	Bird
Porcupine	Warm-blooded	Yes	Yes	Yes	No	No	Mammal
Eel	Cold-blooded	No	No	No	Yes	No	Fish
Salamander	Cold-blooded	No	Yes	Yes	Semi	No	Amphibian

To classify the vertebrates we use the initial state of the two-qubit system as the input attributes and its stationary solution with ${\bar{n}}=0$ as the target attribute. This attribute set includes the properties of a vertebrate such as its body-temperature, skin cover, method of reproduction, ability to fly and ability to live in water which are given in Table 1. Clearly, since the density matrix of a two-qubit system lives in the Hilbert space ${\mathcal {H}} \equiv {\mathbb {C}}^2 \otimes {\mathbb {C}}^2$, therefore its density operator can be introduced by a $4 \times 4$ matrix in terms of tensor products of the single qubit basis $\{{\mathbb {I}}_2 \otimes {\mathbb {I}}_2,{\mathbb {I}}_2 \otimes {\sigma }^i,{\sigma }^i \otimes {\mathbb {I}}_2, {\sigma }^i \otimes {\mathbb {\sigma }}^j \}$ with $i,j=1,2$. Accordingly, the density matrix of any two-qubit system can be parametrized as,

28 $$\begin{aligned} \rho (0)=\frac{1}{4}\left( {\mathbb {I}}_2\otimes {\mathbb {I}}_2+ {\varvec{r}}\cdot \varvec{\sigma }\otimes {\mathbb {I}}_2 + {\mathbb {I}}_2\otimes {\varvec{s}}\cdot \varvec{\sigma } +\sum _{m,n=1}^3 \tau _{m,n} \sigma _m\otimes \sigma _n \right) , \end{aligned}$$

where ${\varvec{r}},{\varvec{s}}\in {\mathbb {R}}^3$ such that $\Vert {\varvec{r}}\Vert \le 1$, $\Vert {\varvec{s}}\Vert \le 1$, and $\varvec{\sigma }=(\sigma _1,\sigma _2,\sigma _3)$ is the vector of Pauli operators and $\tau$ is a $3\times 3$ real matrix. Thus, the density matrix of the two-qubit system possesses 15 free and real parameters which can be chosen as the initial inputs of a classifier. Also, one can easily change the initial state of the two-qubit to meet all mathematical requirement. Anyway, the input attributes can be considered as the initial state of system as below,

29 $$\begin{aligned} \rho (0)=\begin{bmatrix} \rho _{11} &{} \rho _{12} &{} \rho _{13} &{} \rho _{14}\\ \rho _{21} &{} \rho _{22} &{} \rho _{23} &{} \rho _{24}\\ \rho _{31} &{} \rho _{32} &{} \rho _{33} &{} \rho _{34}\\ \rho _{41} &{} \rho _{42} &{} \rho _{43} &{} \rho _{44}\\ \end{bmatrix}=\begin{bmatrix} \rho _{11} &{} \rho _{12} &{} \rho _{13} &{} \rho _{14}\\ \rho _{21} &{} H &{} E &{} W\\ \rho _{31} &{} E &{} L &{} C\\ \rho _{41} &{} W &{} C &{} \rho _{44}\\ \end{bmatrix}, \end{aligned}$$

where $H=2$, $E=1$ and $L=2$ encode the vertebrates that possess these characteristics hibernate, egg-laying and four-legged, respectively. Otherwise, they do not hibernate ($H=1$), do not have four legs ($L=1$), but give birth ($E=0$). Also, $W=1$ ($C=0$) and $C=1$ ($W=0$) denote warm- and cold-blooded creatures. The assigned codes to the considered attributes are given in Table 2. It is worth to note that other elements of right hand side of Eq. (29) do not affect the stationary state of the system, nevertheless they can be adjusted to meet the mathematical requirement of density matrix such as its positivity feature i.e., $|\rho _{ij}|^2\le \rho _{ii}\rho _{jj}$ and normalization condition $\rho _{11}+H+L+\rho _{44}=1$. Here, we emphasize that for simplicity of analysis, we assign integer numbers to each considered code. Thus, a proper normalization factor is needed to satisfy required conditions of the system.

**Table 2:** Attributes and their codes.

Attribute	Code
Hibernate	H=2
No hibernate	H=1
Egg-laying	E=1
Give birth	E=0
Four-legged	L=2
No four-legged	L=1
Warm-blooded	W=1 (C=0)
Cold-blooded	C=1 (W=0)

We calibrate the stationary solutions of the system (Eq. (8)) as below,

30 $$\begin{aligned} P_1= & {} H+L-2E, \nonumber \\ P_2= & {} W-C. \end{aligned}$$

It should be noticed that the steady state solution of the system is independent of the entries which do not occupy by *H*, *E*, *W*, *L*, *C*. Now, we proceed to solve the classification problem, qualitatively. If one intends to achieve the exact solution, all mathematical requirements corresponding to the density operator should be satisfied. Anyway, based on the above values of $P_1$ and $P_2$, we can classify vertebrates as will be described in the continuation. It is clear that $P_2=1$ and $P_2=-1$ denote warm- and cold-blooded species, respectively. Also, one knows that all vertebrates with cold-blooded feature cannot be mammal. Therefore, all the vertebrates with $P_2=-1$ are removed from mammal category. Hence, we should evaluate $P_1$ for all warm-blooded vertebrates which previously identified by $P_2=1$. In this regard, we define a decision boundary to separate mammal from non-mammal vertebrates. For instance $P_1=2$ is a proper decision boundary. Therefore, we can classify vertebrates as two classes of mammal ($P_1>2$) and non-mammal ($P_1 \le 2$) which are referred as the first (M) and second (N) classes i.e. a binary classification problem.

The results are presented in Table 3. A comparison with the vertebrate data set presented in Table 1 shows that both human and whale are misclassified with this decision boundary ($P_1=2$). Since both of them are mammals, while they are classified as non-mammal by this classifier. Generally, the results reveal that two of sixteen training records are mislabeled i.e., human and whale were classified as non-mammal instead of mammals. This is a good result with $87.5\%$ accuracy, in comparison with other classifiers such as decision tree and so on. The classification of vertebrates with decision tree algorithm was already done in literature.

**Figure 4:**
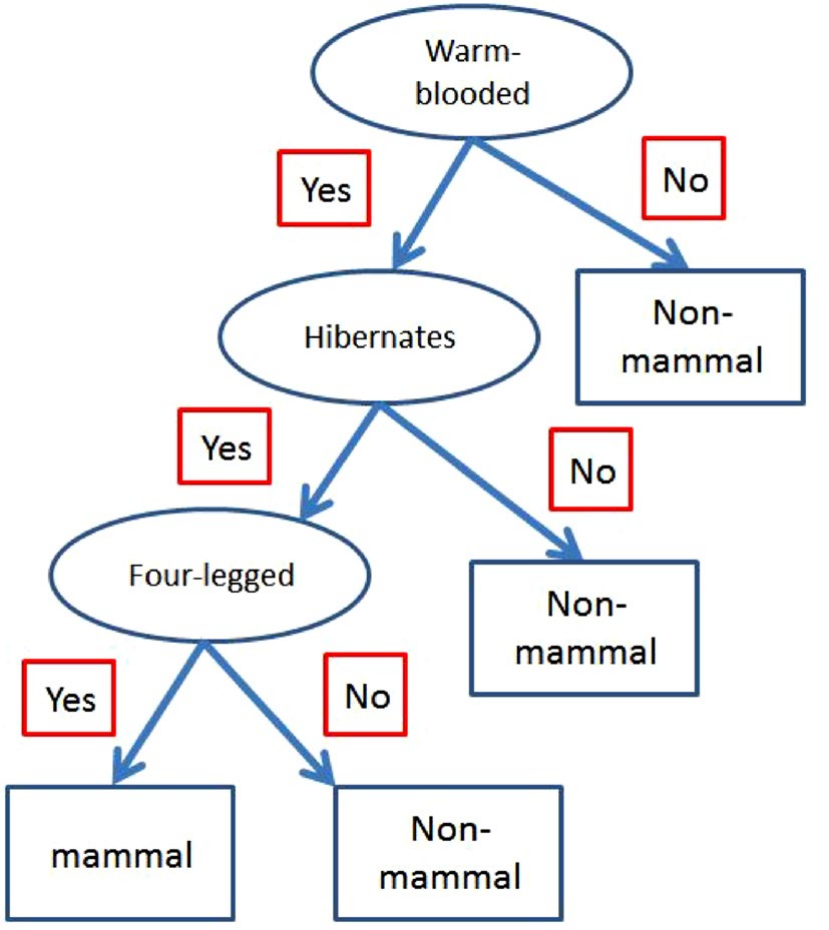
A typical decision tree algorithm for classification of vertebrates.

To show the accuracy of our classifier with respect to decision tree, we present the result of the latter classifier in the last column of Table 3 based on a typical algorithm shown in Fig. 4. As clearly can be observed, this algorithm classifies all warm-blooded vertebrates that do not hibernates as non-mammal. Thus, human, whale and cat are misclassified. Moreover, bat has only two legs, therefore it is misclassified as non-mammal. In conclusion, the accuracy of decision tree algorithm is $75\%$ which is lower than the accuracy of our model.

Meanwhile, it is worth to emphasize that, our two-qubit encoding does not require any iterative procedure to improve the classifier’s performance. Naturally, errors due to exceptional records are always unavoidable and establish the minimum error rate achievable by any classifier. Moreover, we can improve the accuracy of our classifier by assigning $P \ge 3$ to mammals and $P<3$ to non-mammals where $P=P_1+P_2$. The classification of vertebrates based on these new class labels shows that human and whale are truly categorized as mammal and the accuracy of classifier can be improved. Briefly, both classifiers can be encoded by the following relations,

31 $$\begin{aligned} P_2= & {} \left\{ \begin{array}{cc} 1 &{} \quad {\mathrm{warm-blooded}} \\ -1 &{} \quad {\mathrm{cold-blooded}} \\ \end{array} \right. \nonumber \\ P_1= & {} \left\{ \begin{array}{cc} > 2 &{} \quad {\mathrm{mammal }}\quad {\mathrm{if }}\quad {\mathrm{P_2=1 }} \\ \le 2 &{} \quad { \mathrm non-mammal} \\ \end{array} \right. \nonumber \\ P= & {} \left\{ \begin{array}{cc} P_1+P_2 \ge 3 &{} \quad {\mathrm{mammal}} \\ P_1+P_2 < 3 &{} \quad {\mathrm{non-mammal}} \\ \end{array} \right. \end{aligned}$$

Again, we mention that the first classifier identifies a mammal vertebrate ($P_1 > 2$), once it is a warm-blooded creature ($P_2=1$). Also, it is worth to note that besides the simplicity of the second classifier, it generally presents higher accuracy with respect to the first one. Furthermore, one can predict the class labels of other species of vertebrates with our proposed classifiers. For instance, suppose that we are given the characteristics of two creatures known as flamingo and gila monster as shown in Table 4 and we want to predict their class labels. Both classifiers predict that these creatures are non-mammal vertebrates as one can confirm our true prediction. The results are shown in Table 4, both flamingo and gila monster are assigned $P_1 \le 2$ and $P<3$ based on the first and second classifiers, respectively. Finally, it is worth to pay our attention to the role of quantum effect (entanglement) in machine learning. It has been shown that there exist some scenarios wherein entanglement between different neurons of a neural network is needed to perform some special tasks^55^. In our scheme, the interaction between the two-qubit system and environment leads to the entanglement between the initial inputs (elements of density matrix) which can be considered as a combination (or weighed sum) of the entries of a neural network.

**Table 3:** The results of classification of vertebrates with two quantum classifiers (QCs) and decision tree algorithm as a classical one.

Record	W	E	L	H	QC	$P_1$	$P_2$	P	DT
Human	1	0	1	1	N*	2	1	3	N*
Python	0	1	1	2	N	1	−1	0	N
Salmon	0	1	1	1	N	0	−1	−1	N
Whale	1	0	1	1	N*	2	1	3	N*
Frog	0	1	2	2	N	2	−1	1	N
Komodo dragon	0	1	2	1	N	1	−1	0	N
Bat	1	0	1	2	M	3	1	4	N*
Cat	1	0	2	1	M	3	1	4	N*
Leopard shark	0	0	1	1	N	2	−1	1	N
Turtle	0	1	2	1	N	1	−1	0	N
Penguin	1	1	1	1	N	0	1	1	N
Porcupine	1	0	2	2	M	4	1	5	M
Eel	0	1	1	1	N	0	−1	−1	N
Salamander	0	1	2	2	N	2	−1	1	N
Eagle	1	1	1	1	N	0	−1	−1	N
Guppy	0	0	1	1	N	2	−1	1	N

The results of the first quantum classifier are presented in 6th column. Here, $P_1>2$ and $P_2=1$ identify mammal vertebrates. The mammals (M) and non-mammal (N) vertebrates can be classified via the second classifier based on the values of *P* as shown in 9th column where mammal creatures possess $P\ge 3$. The first classifier presents two misclassified records, while the second one correctly classifies all of them. The results of decision tree (DT) algorithm are presented in th last column. The misclassified records are marked with asterisk symbol (*).

**Table 4:** Prediction of class labels.

Name	Body temperature	Give birth	Four-legged	Hibernates	Mammal	$P_1$	$P_2$	P
Flamingo	Warm-blooded	No	No	No	?	0	1	1
Gila monster	Cold-blooded	No	Yes	Yes	?	2	−1	1

## Summary and conclusions

We investigated the generation of stationary entangled state via considering a two-qubit system interacting with a thermal environment. For this reason, we analytically solved the quantum master equation and found its steady state solution. A class of stationary entangled states such as the Werner-like and MEMSs were generated in the steady state regime. Also, we showed that one can manipulate the initial state of the two-qubit system to construct robust Bell-like state. Next, the population and coherence of the considered two-qubit system were investigated and we demonstrated that the system presents the residual coherence even in the equilibrium condition. In the continuation, as an interesting and practical application of open quantum system, we encoded the solution of the two-qubit system to solve a binary classification. To achieve the purpose, we classified the vertebrates into two groups; mammals and non-mammals. In spite of its simplicity and without requiring any iterative procedure, our system solved this binary classification problem with high enough accuracy. Also, our classifiers can be used to predict the class label of other unknown vertebrates. Moreover, we showed that the quantum classifiers based on the two-qubit system outperform the classical algorithms i.e, the decision tree classifier.

## Appendix A: Matrix representation of Lindblad operator

The matrix *M* with respect to the basis $\{{|{1}\rangle },{|{2}\rangle },{|{3}\rangle },{|{4}\rangle }\}$ in (34) can be obtained as below:

32 $$\begin{aligned} M=\begin{bmatrix} M_{11}&{} M_{12} \\ M_{21} &{} M_{22} \end{bmatrix}, \end{aligned}$$

where

$$\begin{aligned} M_{11}= & {} \begin{bmatrix} -4\bar{n}-4 &{} 0 &{} 0 &{} 0 &{} 0 &{} 0 &{} 0 &{} 0\\ 0 &{} -4\bar{n}-3 &{} -\bar{n}-1 &{} 0 &{} 0 &{} 0 &{} 0 &{} 2\bar{n}\\ 0 &{} -2\bar{n}-1 &{} -4\bar{n}-3 &{} 0 &{} 0 &{} 0 &{} 0 &{} 2\bar{n}\\ 0 &{} 0 &{} 0 &{} -4\bar{n}-2 &{} 0 &{} 0 &{} 0 &{} 0\\ 0 &{} 0 &{} 0 &{} 0 &{} -4\bar{n}-3 &{} 0 &{} 0 &{} 0\\ 2\bar{n}+2 &{} 0 &{} 0 &{} 0 &{} 0 &{} -4\bar{n}-2 &{} -2\bar{n}-1 &{} 0\\ 2\bar{n}+2 &{} 0 &{} 0 &{} 0 &{} 0 &{} -2\bar{n}-1 &{} -4\bar{n}-2 &{} 0\\ 0 &{} 2\bar{n}+2 &{} 2\bar{n}+2 &{} 0 &{} 0 &{} 0 &{} 0 &{} -4\bar{n}-1\\ \end{bmatrix}\\ M_{12}= & {} \begin{bmatrix} 0 &{} 2\bar{n} &{} 2\bar{n} &{} 0 &{} 0 &{} 0 &{} 0 &{} 0\\ 0 &{} 0 &{} 0 &{} 2\bar{n} &{} 0 &{} 0 &{} 0 &{} 0\\ 0 &{} 0 &{} 0 &{} 2\bar{n} &{} 0 &{} 0 &{} 0 &{} 0\\ 0 &{} 0 &{} 0 &{} 0 &{} 0 &{} 0 &{} 0 &{} 0\\ -2\bar{n}-1 &{} 0 &{} 0 &{} 0 &{} 0 &{} 2\bar{n} &{} 2\bar{n} &{} 0\\ 0 &{} -2\bar{n}-1 &{} 0 &{} 0 &{} 0 &{} 0 &{} 0 &{} 2\bar{n}\\ 0 &{} 0 &{} -2\bar{n}-1 &{} 0 &{} 0 &{} 0 &{} 0 &{} 2\bar{n}\\ 0 &{} 0 &{} 0 &{} -2\bar{n}-1 &{} 0 &{} 0 &{} 0 &{} 0\\ \end{bmatrix}\\ M_{21}= & {} \begin{bmatrix} 0 &{} 0 &{} 0 &{} 0 &{} -2\bar{n}-1 &{} 0 &{} 0 &{} 0\\ 2\bar{n}+2 &{} 0 &{} 0 &{} 0 &{} 0 &{} -2\bar{n}-1 &{} 0 &{} 0\\ 2\bar{n}+2 &{} 0 &{} 0 &{} 0 &{} 0 &{} 0 &{} -2\bar{n}-1 &{} 0\\ 0 &{} 2\bar{n}+2 &{} 2\bar{n}+2 &{} 0 &{} 0 &{} 0 &{} 0 &{} -2\bar{n}-1\\ 0 &{} 0 &{} 0 &{} 0 &{} 0 &{} 0 &{} 0 &{} 0\\ 0 &{} 0 &{} 0 &{} 0 &{} 2\bar{n}+2 &{} 0 &{} 0 &{} 0\\ 0 &{} 0 &{} 0 &{} 0 &{} 2\bar{n}+2 &{} 0 &{} 0 &{} 0\\ 0 &{} 0 &{} 0 &{} 0 &{} 0 &{} 2\bar{n}+2 &{} 2\bar{n}+2 &{} 0\\ \end{bmatrix}\\ M_{22}= & {} \begin{bmatrix} -4\bar{n}-3 &{} 0 &{} 0 &{} 0 &{} 0 &{} 2\bar{n} &{} 2\bar{n} &{} 0\\ 0 &{} -4\bar{n}-2 &{} -2\bar{n}-1 &{} 0 &{} 0 &{} 0 &{} 0 &{} 2\bar{n}\\ 0 &{} -2\bar{n}-1 &{} -4\bar{n}-2 &{} 0 &{} 0 &{} 0 &{} 0 &{} 2\bar{n}\\ 0 &{} 0 &{} 0 &{} -4\bar{n}-1 &{} 0 &{} 0 &{} 0 &{} 0\\ 0 &{} 0 &{} 0 &{} 0 &{} -4\bar{n}-2 &{} 0 &{} 0 &{} 0\\ 2\bar{n}+2 &{} 0 &{} 0 &{} 0 &{} 0 &{} -4\bar{n}-1 &{} -4\bar{n}-1 &{} 0\\ 2\bar{n}+2 &{} 0 &{} 0 &{} 0 &{} 0 &{} -2\bar{n}-1 &{} -2\bar{n}-1 &{} 0\\ 0 &{} 2\bar{n}+2 &{} 2\bar{n}+2 &{} 0 &{} 0 &{} 0 &{} 0 &{} -4\bar{n}\\ \end{bmatrix} \end{aligned}$$

In fact, the matrix *M* plays the role of Lindblad operator in matrix representation as can be found from the comparison of Eq. (33) with Eq. (36). Therefore, to get $\ker (\mathcal {L})$ one can use $\ker (M)$. For $\bar{n}=0$, the dimension of $\ker (M)$ is 4 and a basis is given by:

33 $$\begin{aligned} k_1=\begin{bmatrix} 0 &{} 0 &{} 0 &{} 0\\ 0 &{} 0 &{} 0 &{} 0\\ 0 &{} 0 &{} 0 &{} 0\\ 0 &{} 0 &{} 0 &{} 1\\ \end{bmatrix},\quad k_2=\begin{bmatrix} 0 &{} 0 &{} 0 &{} 0\\ 0 &{} 0 &{} 0 &{} 0\\ 0 &{} 0 &{} 0 &{} 0\\ 0 &{} -1 &{} 1 &{} 0\\ \end{bmatrix},\quad k_3=\begin{bmatrix} 0 &{} 0 &{} 0 &{} 0\\ 0 &{} 0 &{} 0 &{} -1\\ 0 &{} 0 &{} 0 &{} 1\\ 0 &{} 0 &{} 0 &{} 0\\ \end{bmatrix},\quad k_4=\begin{bmatrix} 0 &{} 0 &{} 0 &{} 0\\ 0 &{} 1 &{} -1 &{} 0\\ 0 &{} -1 &{} 1 &{} 0\\ 0 &{} 0 &{} 0 &{} 0\\ \end{bmatrix}. \end{aligned}$$

Instead, for $\bar{n} > 0$, the dimension of *ker*(*M*) is 2 and a basis is given by:

34 $$\begin{aligned} k'_1=\begin{bmatrix} 0 &{} 0 &{} 0 &{} 0\\ 0 &{} 0 &{} 0 &{} 0\\ 0 &{} 0 &{} 0 &{} 0\\ 0 &{} -1 &{} 1 &{} 0\\ \end{bmatrix}, \quad k'_2=\begin{bmatrix} 0 &{} 0 &{} 0 &{} 0\\ 0 &{} 1 &{} -1 &{} 0\\ 0 &{} -1 &{} 1 &{} 0\\ 0 &{} 0 &{} 0 &{} 0\\ \end{bmatrix}, \end{aligned}$$

## Appendix B

The solution of dynamical evolution of system Eq. (36) for $\bar{n}=0$ can be obtained as follows,

35 $$\begin{aligned} \rho _{11}(t)= & {} \rho _{11} e^{-4t}, \nonumber \\ \rho _{12}(t)= & {} \rho _{21}^*(t)=\frac{1}{2}(\rho _{12}+\rho _{13})e^{-4t}+\frac{1}{2}(\rho _{12}-\rho _{13})e^{-2t}, \nonumber \\ \rho _{13}(t)= & {} \rho _{31}^*(t)=\frac{1}{2}(\rho _{12}+\rho _{13})e^{-4t}-\frac{1}{2}(\rho _{12}-\rho _{13})e^{-2t} \nonumber \\ \rho _{14}(t)= & {} \rho _{41}^*(t)=\rho _{14} e^{-2t}, \nonumber \\ \rho _{22}(t)= & {} \rho _{33}(t)= (\frac{3}{2}+2t) \rho _{11} e^{-4t}+\frac{1}{8}(-12\rho _{11}+2\rho _{22}+2\rho _{23}+2\rho _{32})e^{-4t} -\frac{1}{2}(-\rho _{22}+\rho _{33})e^{-2t} \nonumber \\ {}+ & {} \frac{1}{4}(\rho _{22}+\rho _{33}-\rho _{23}-\rho _{32}), \nonumber \\ \rho _{23}(t)= & {} \rho _{32}^*(t)= (\frac{3}{2}+2t) \rho _{11} e^{-4t}+\frac{1}{8}(-12\rho _{11}+2\rho _{22}+2\rho _{23}+2\rho _{32})e^{-4t} -\frac{1}{2}(-\rho _{22}+\rho _{33})e^{-2t} \nonumber \\ {}+ & {} \frac{1}{4}(-\rho _{22}-\rho _{33}+\rho _{23}+\rho _{32}), \nonumber \\ \rho _{24}(t)= & {} \rho _{42}^*(t)= (\rho _{12}-\rho _{13})(e^{-4t}-2e^{-2t})+\frac{1}{2}(6\rho _{12}-2\rho _{13}+\rho _{24}+\rho _{34})e^{-2t}+\frac{1}{2}(\rho _{24}-\rho _{34}), \nonumber \\ \rho _{34}(t)= & {} \rho _{43}^*(t)= (\rho _{12}-\rho _{13})(e^{-4t}-2e^{-2t})+\frac{1}{2}(6\rho _{12}-2\rho _{13}+\rho _{24}+\rho _{34})e^{-2t}-\frac{1}{2}(\rho _{24}-\rho _{34}), \nonumber \\ \rho _{44}(t)= & {} 1-(\rho _{11}(t)+2\rho _{22}(t)). \end{aligned}$$

## Appendix C

36 $$\begin{aligned} f_{11}(\bar{n})= & {} \sqrt{\bar{n}(\bar{n}+1)}(8\bar{n}^5+12\bar{n}^4+12\bar{n}^3+4\bar{n}^2)+8\bar{n}^6+16\bar{n}^5+8\bar{n}^4, \nonumber \\ f_{12}(\bar{n})= & {} \sqrt{\bar{n}(\bar{n}+1)}(4\bar{n}^5+6\bar{n}^4+6\bar{n}^3+2\bar{n}^2)+4\bar{n}^6+8\bar{n}^5+4\bar{n}^4, \nonumber \\ g_1(\bar{n})= & {} \frac{1}{4}\big [ \sqrt{\bar{n}(\bar{n}+1)}(3\bar{n}^3+6\bar{n}^2+4\bar{n}+1)+3\bar{n}^4+3\bar{n}^3+\bar{n}^2)(\bar{n}^2+2\bar{n}+1+\bar{n}\sqrt{\bar{n}(\bar{n}+1)}\big ], \end{aligned}$$

37 $$\begin{aligned} f_{21}(\bar{n})= & {} -\big [\sqrt{\bar{n}(\bar{n}+1)}(8\bar{n}^5+20\bar{n}^4+24\bar{n}^3+16\bar{n}^2+4\bar{n})+8\bar{n}^6+24\bar{n}^5+24\bar{n}^4+8\bar{n}^3\big ], \nonumber \\ f_{22}(\bar{n})= & {} -\big [\sqrt{\bar{n}(\bar{n}+1)}(16\bar{n}^5+40\bar{n}^4+52\bar{n}^3+38\bar{n}^2+14\bar{n}+2) \nonumber \\&\quad +16\bar{n}^6+48\bar{n}^5+52\bar{n}^4+24\bar{n}^3+4\bar{n}^2\big ], \nonumber \\ f_{23}(\bar{n})= & {} \big [\sqrt{\bar{n}(\bar{n}+1)}(8\bar{n}^5+20\bar{n}^4+28\bar{n}^3+22\bar{n}^2+10\bar{n}+2)+8\bar{n}^6+24\bar{n}^5+28\bar{n}^4+16\bar{n}^3+4\bar{n}^2\big ], \nonumber \\ g_2(\bar{n})= & {} -\frac{1}{8}(\bar{n}^2+2\bar{n}+1+\bar{n}\sqrt{\bar{n}(\bar{n}+1)})(\bar{n}^2+(\bar{n}+1)\sqrt{\bar{n}(\bar{n}+1)})(4\bar{n}^2+3\bar{n}), \end{aligned}$$

38 $$\begin{aligned} f_{31}(\bar{n})= & {} -\big [\sqrt{\bar{n}(\bar{n}+1)}(8\bar{n}^5+20\bar{n}^4+24\bar{n}^3+16\bar{n}^2+4\bar{n})+8\bar{n}^6+24\bar{n}^5+24\bar{n}^4+8\bar{n}^3\big ], \nonumber \\ f_{32}(\bar{n})= & {} \big [\sqrt{\bar{n}(\bar{n}+1)}(8\bar{n}^5+20\bar{n}^4+28\bar{n}^3+22\bar{n}^2+10\bar{n}+2)+8\bar{n}^6+24\bar{n}^5+28\bar{n}^4+16\bar{n}^3+4\bar{n}^2\big ], \nonumber \\ f_{33}(\bar{n})= & {} -\big [\sqrt{\bar{n}(\bar{n}+1)}(16\bar{n}^5+40\bar{n}^4+52\bar{n}^3+38\bar{n}^2+14\bar{n}+2) \nonumber \\&\quad +16\bar{n}^6+48\bar{n}^5+52\bar{n}^4+24\bar{n}^3+4\bar{n}^2\big ]. \end{aligned}$$
